# POD–Kalman filtering for improving noninvasive 3D temperature monitoring in MR‐guided hyperthermia

**DOI:** 10.1002/mp.15811

**Published:** 2022-06-26

**Authors:** Iva VilasBoas‐Ribeiro, Sven A.N. Nouwens, Sergio Curto, Bram de Jager, Martine Franckena, Gerard C. van Rhoon, W. P. M. H. Heemels, Margarethus M. Paulides

**Affiliations:** ^1^ Department of Radiotherapy Erasmus MC University Medical Center Rotterdam The Netherlands; ^2^ Control System Technology Group Department of Mechanical Engineering Eindhoven University of Technology Eindhoven The Netherlands; ^3^ Department of Radiation Science and Technology Faculty of Applied Sciences Delft University of Technology Delft The Netherlands; ^4^ Care and Cure Research Lab (EM‐4C&C) of the Electromagnetics Group Department of Electrical Engineering Eindhoven University of Technology Eindhoven The Netherlands

**Keywords:** hyperthermia, Kalman filter, MR thermometry, proper orthogonal decomposition, thermotherapy

## Abstract

**Background:**

During resonance frequency (RF) hyperthermia treatment, the temperature of the tumor tissue is elevated to the range of 39–44°C. Accurate temperature monitoring is essential to guide treatments and ensure precise heat delivery and treatment quality. Magnetic resonance (MR) thermometry is currently the only clinical method to measure temperature noninvasively in a volume during treatment. However, several studies have shown that this approach is not always sufficiently accurate for thermal dosimetry in areas with motion, such as the pelvic region. Model‐based temperature estimation is a promising approach to correct and supplement 3D online temperature estimation in regions where MR thermometry is unreliable or cannot be measured. However, complete 3D temperature modeling of the pelvic region is too complex for online usage.

**Purpose:**

This study aimed to evaluate the use of proper orthogonal decomposition (POD) model reduction combined with Kalman filtering to improve temperature estimation using MR thermometry. Furthermore, we assessed the benefit of this method using data from hyperthermia treatment where there were limited and unreliable MR thermometry measurements.

**Methods:**

The performance of POD–Kalman filtering was evaluated in several heating experiments and for data from patients treated for locally advanced cervical cancer. For each method, we evaluated the mean absolute error (MAE) concerning the temperature measurements acquired by the thermal probes, and we assessed the reproducibility and consistency using the standard deviation of error (SDE). Furthermore, three patient groups were defined according to susceptibility artifacts caused by the level of intestinal gas motion to assess if the POD–Kalman filtering could compensate for missing and unreliable MR thermometry measurements.

**Results:**

First, we showed that this method is beneficial and reproducible in phantom experiments. Second, we demonstrated that the combined method improved the match between temperature prediction and temperature acquired by intraluminal thermometry for patients treated for locally advanced cervical cancer. Considering all patients, the POD–Kalman filter improved MAE by 43% (filtered MR thermometry = 1.29°C, POD–Kalman filtered temperature = 0.74°C). Moreover, the SDE was improved by 47% (filtered MR thermometry = 1.16°C, POD–Kalman filtered temperature = 0.61°C). Specifically, the POD–Kalman filter reduced the MAE by approximately 60% in patients whose MR thermometry was unreliable because of the great amount of susceptibilities caused by the high level of intestinal gas motion.

**Conclusions:**

We showed that the POD–Kalman filter significantly improved the accuracy of temperature monitoring compared to MR thermometry in heating experiments and hyperthermia treatments. The results demonstrated that POD–Kalman filtering can improve thermal dosimetry during RF hyperthermia treatment, especially when MR thermometry is inaccurate.

## 1 INTRODUCTION

Hyperthermia is an adjuvant cancer treatment that enhances the effects of radiotherapy and chemotherapy by locally applying heat to the tumor region.^1^, ^2^ A retrospective analysis done by Franckena et al.^3^ showed that in 420 patients with locally advanced carcinoma, the probability of cure is correlated with the administered thermal dose.^4^ That study illustrated the clinical relevance of careful temperature monitoring and the need to optimize thermal dose. However, even though intraluminal thermometry is the golden standard for temperature assessment during treatment,^5^, ^6^ it has severe limitations like information from only a few locations.^7^, ^8^ The combination of a magnetic resonance (MR)‐compatible hyperthermia device with MR imaging might be the ideal technology since it enables to monitor temperature noninvasively, achieve dose‐optimization in real time, and perform real‐time quality assurance.^9^, ^10^ MR thermometry is currently the only clinical option to measure temperature noninvasively; however, it is prone to noise and motion artifacts, and its accuracy in the pelvic region is insufficient for real‐time thermal dosimetry. Hence, new developments are urgently needed to improve MR thermometry measurements^11^, ^12^, ^13^ and enable thermal dosimetry during treatment. A combined proper orthogonal decomposition (POD)–Kalman filtering method was recently proposed,^14^ but its performance in experimental and in in vivo settings has not been rigorously studied. To establish its potential, we studied the reproducibility of this method using several heating experiments. Most importantly, we explored the potential of POD–Kalman filtering to reduce the sensitivity to motion artifacts and enable the real‐time temperature monitoring using in vivo data from hyperthermia treatments.

Noninvasive monitoring of temperature is possible using several temperature‐sensitive MR parameters. The most used MR method is the proton resonance frequency shift (PRFS),^15^, ^16^, ^17^ which measures relative temperature differences based on the phase change. Several studies have demonstrated the feasibility of the PRFS method in both phantoms and patients.^12^, ^18^ The study conducted by Curto et al.^9^ showed that MR thermometry accuracy in phantoms was between 0.3°C and 0.5°C. Gellermann et al.^19^ showed the potential of MR thermometry in patients with recurrent rectal carcinoma and found accuracies of 1.5°C using thermistor probes as the gold standard. Amid the different studies that evaluated MR thermometry in a group of patients, motion and B0 field changes appeared as the main problem in achieving accurate MR thermometry measurements.^11^, ^20^, ^21^ Feddersen et al. showed that reliable MR thermometry measurements are not being achieved in regions with motion, regardless of the MR method.^11^ Our recent work confirmed that intestinal gas motion caused strong susceptibilities artifacts in MR thermometry, and that its quantification prior to treatment predicts MR thermometry accuracy during treatment.^22^ Moreover, the number of MR thermometry updates is restricted during treatment due to the water circulation through the water bolus surrounding the patient. Because water circulation creates significant susceptibility artifacts, water circulation has to be stopped for a certain period before an MR thermometry acquisition can be taken. Hence, the inaccuracies of MR thermometry during hyperthermia treatment must be solved, but there is also a strong need for temperature information between scans.

Various studies have been investigating model‐based temperature estimation to enhance temperature measurements. Potocki et al.^23^ proposed Kalman filtering to estimate the temperature in unmeasured locations.^24^ They used the bio‐heat transfer equation (BHTE) model and temperature information acquired with invasive probes.^25^ For high‐intensity focused ultrasound (HIFU), several studies employed filtering in combination with MR thermometry to improve the temperature estimation.^26^, ^27^ Roujol et al.^28^ employed a Kalman filter combined with MR thermometry and showed a threefold accuracy improvement for temperature estimation when heating a phantom and a porcine kidney. Zhang et al.^29^ and Schmitt et al.^30^ have shown combining Kalman filtering with MR thermometry to improve the temperature monitoring accuracy on patient data using HIFU. Note that HIFU focuses on heating a volume up to 1.8 cm in diameter and, around 2.4 cm in length^31^, ^32^, ^33^; thus, the region of interest (ROI) used for temperature monitoring can be much smaller, that is, less than 20 × 20 cm^2^,^33^, ^34^ which strongly simplifies the thermal modeling. Overall, extensive studies using this method in patient data or larger regions were not performed.

The treatment region for resonance frequency (RF) hyperthermia in pelvic tumors is approximately 14 cm and 25.5 cm in diameter and length.^35^ Additionally, the ROI for MR thermometry is 50 × 50 cm^2^ since the RF antennas are placed in circular arrays around the patient, and near field hotspots, while lower, can form all around. Due to increased computational complexity, the extensive state‐space systems resulting from the large field of view (FOV) prohibit online Kalman‐filtering techniques. Therefore, to enable Kalman‐filtering for RF‐hyperthermia, adequate reduced‐order models are needed that enable fast computation. Hendrikx et al.^14^ validated the use of reduced thermal models for recursive temperature estimation using POD^36^ of MR thermometry in phantoms. In that study, the precomputation of patient‐specific heating modes enabled accurate and efficient models that captured the complexity of the geometry without compromising spatial resolution. Even though the feasibility of the POD–Kalman filter was shown, this study did not systematically evaluate reproducibility. Furthermore, no research has shown the benefit of the POD–Kalman filter to improve temperature prediction of MR thermometry for large volumes when there are limited and unreliable MR thermometry measurements. Moreover, the feasibility and clinical potential of POD–Kalman filtering during RF hyperthermia was not shown for patient data with complex modeling and unreliable MR thermometry.

This retrospective study presents the reproducibility of temperature estimation in heating experiments and provides the first feasibility study of this method in patient data. As such, we validated combining reduced models and MR thermometry to make the best estimate of the temperature. Our study evaluates the benefit of this method to enable real‐time temperature monitoring when MR thermometry measurements are not reliable. Hence, the performance of the POD–Kalman filter was also specifically analyzed for correcting severe artifacts and when limited MR thermometry measurements are available.

## 2 MATERIALS AND METHODS

### 2.1 MR thermometry acquisition and processing

For temperature monitoring, we employed the proton resonance frequency shift (PRFS) method.^13^, ^15^, ^16^ We used the clinical sequence part of a Conformité Européene (CE) ‐marked MR thermometry imaging package provided by the manufacturer Dr. Sennewald Medizintechnik GmbH (Munchen, Germany). This sequence is a double echo gradient recalled echo (DEGRE) sequence^15^ with parameters: echo times: TE = 4.8 and 19.1 ms; repetition time: TR = 620 ms; 25 axial slices; slice thickness = 1 cm; no separation between slices; FOV = 50 cm × 50 cm; acquisition matrix = 128 × 128; reconstruction matrix = 256 × 256; flip angle = 40°; scan time = 83 s.

The PRFS method measures relative temperature differences (ΔT) based on phase changes of the different MR thermometry scans.^16^, ^37^ Before switching the power of the heating system on, the PRFS sequence acquisition is started such a reference phase data (φ_n0_) is acquired in baseline conditions. During treatment, multiple phase data sets are acquired ($\varphi_{n})$. The raw MR thermometry was calculated by subtracting the phase images according to Equation (1).

(1) $$\Delta T\;\left(n\right)=\frac{\varphi_{n}-\;\varphi_{n0}}{\gamma \alpha B_{0}TE},$$

where γ is the gyromagnetic ratio equal to 267.5 ×10^6^; α is the PRF‐thermal coefficient for aqueous tissue, and it is equal to −0.01 ppm/°,^16^, ^38^, ^39^ which is based on the linear relationship between the PRFS of water and the corresponding temperature change in different types of high water content tissues; *B*_0_ is the magnetic field strength equal to 1.5T; *TE* is the echo time equal to 19.1 ms, *n* is the scanning time, and *n*0 indicates the first scanning time before treatment (baseline condition). A confounder of MR thermometry when using the PRFS method is the B_0_ field drift due to hardware instabilities or gradient coil‐heating during scanning.^40^ It is crucial to correct since the effect is in the order of the RF shift with temperature. The standard clinical method is to correct the drift using fat‐tube references from the MR‐compatible device. Because the fat‐tube references are placed in the periphery of the FOV, these regions suffer from low SNR. Earlier, we found that correction based on the combination of fat‐tube references and the patient's subcutaneous fat improves measurement accuracy.^22^ Hence, we combined the signals in the fat references and subcutaneous fat and employed a 2D polynomial spatial–temporal correction^16^, ^20^, ^41^ across the MR temperature maps to remove changes in PRFS signal in all fat regions throughout the treatment. We consider the error caused by a possible temperature increase in the fat regions to be minor because protons in fat do not exhibit a temperature‐dependent frequency shift.^42^ Moreover, the heating induced by RF heating is generally small compared to tissues with higher water content, and the fat regions used for correction are not near the heated regions. After the B0 drift correction, these temperature maps were defined as MR thermometry.

### 2.2 Patient and phantom modeling

All measurements were conducted in the BSD‑2000‑3D Sigma Eye MR‑compatible system^9^, ^19^ (Pyrexar Medical Corp., Salt Lake City, UT, USA) integrated into a 1.5T GE system (GE Discovery MR450w General Electric, Milwaukee, WI, USA). Note that the patient and phantom model generation was conducted after the heating experiments and hyperthermia treatments. The duration of the patient and phantom modeling was approximately 2 h.

We used two identical anthropomorphous phantoms filled with muscle‐equivalent material^9^ and plastic material resembling the human pelvis, spine bones, and the discs of a skeleton.^9^, ^14^ Hence, the phantoms mimic the shape and dielectric and thermal properties of the trunk of a human body, and have a total length of 60 cm and a weight of 30 kg, respectively. Before the heating experiments, computed tomography (CT) scans were taken. The different phantom tissues (muscle‐equivalent filling, bone plastic and outer shell plastic) were delineated using iSeg (v3.10 Zurich MTech AG, Zurich, Switzerland), and afterward, a full 3D phantom model was generated. To reproduce the heating experiments in the simulations, we used the software package Sim4Life (v5.2 Zurich MedTech AG, Zurich, Switzerland), to position the 3D phantom model inside the 3D model of the hyperthermia device according to the MR images taken during the experiment.^9^, ^12^

The 14 patients included in this retrospective study were diagnosed with locally advanced cervical carcinoma. Moreover, all patients were treated with curative intent using hyperthermia as an adjuvant to radiotherapy, and these treatments were conducted between 2017 and 2019. All patients included were female subjects with an average age of 56.7 ± 16.7 years who had a histologically confirmed cervical carcinoma. We included patients with various FIGO stages: IA (one patient), IB (two patients), IIB (five patients), IIIB (four patients), and IVA (two patients). The medical ethics committee approved the research protocol for this investigation of Erasmus MC, University Medical Center Rotterdam. Note that the approval was obtained prior to the start of the study, and the ethics code is MEC 2015–108. Additionally, we have received informed consent from all the patients included in this study.

**FIGURE 1:**
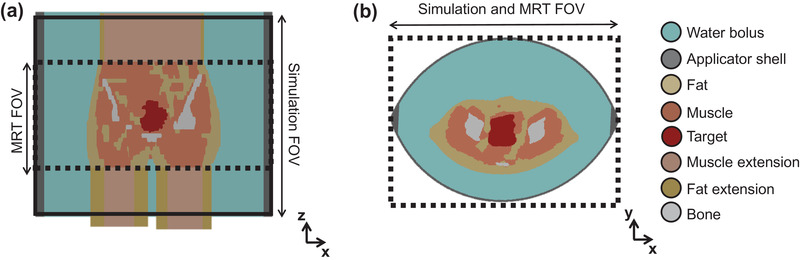
Schematic representation of a patient model and its position in the hyperthermia device. The simulation and MR thermometry FOV are displayed in the coronal (a) and axial (b) views. In the axial view, the FOVs are overlapped

The modeling procedure was equal to the phantom study, except that we used the MR images taken at the start of treatment with the patient in treatment position for tissue delineation instead of the CT images. Four tissue types were delineated: fat, muscle, bone, and target volume.^43^, ^44^, ^45^ The latter structure was used for hyperthermia treatment planning optimization and delineated by a radiation oncologist. The MR thermometry FOV included only 25 cm of the total patient volume inside the hyperthermia applicator (Figure 1). This limited FOV would be inadequate for precise electromagnetic field representation since the water bolus would replace the missing volume of the patient model. For that reason, as presented in Figure 1, fat, bone, and muscle were extended to 50 cm by repeating the first and last slice.^46^ Even though the extended volume does not fully represent the anatomy, we believe that the effect of these errors in the target region will be small. As mentioned before, the focus size in the z‐direction is a maximum of 25 cm; thus, the energy deposition in the border regions will be low. Since the border regions correspond to the extended regions, we believe that the effect of incorrect anatomy will be minor.

### 2.3 Electromagnetic and thermal modeling

The electromagnetic propagation in the 3D models was predicted using finite‐difference time‐domain (FDTD) solver in Sim4Life. A nonuniform grid was used in the simulations, in which the maximum grid step inside and outside of the applicator was 2.5 mm and 10 cm, respectively. The electromagnetic simulations took approximately 3 h, and the resulting 3D EM field distributions were imported into MATLAB. The specific absorption rate (SAR) was calculated using the power and phase settings applied in the experiments and treatments. The antenna settings used in the hyperthermia treatments were optimized using our in‐house treatment planning software (VEDO).^47^

The 3D temperature distribution was calculated using the Pennes' bio‐heat equation (PBHE)^25^:

(2) $$\rho c\;\frac{\partial T}{\partial t}=\nabla \left(k\;\nabla T\right)+\rho Q_{m}+\rho S-\rho_{b}c_{b}\rho \omega \;(T-T_{b}),$$

where *T* (°C) is the temperature, *t* (min) is the time, *c* (J $k{g^{-1}}^{\circ }C^{-1}$) is the specific heat capacity, ρ(kg m^-3^) is the volume density of mass, *k* ($W\;{m^{-1}}^{\circ }C^{-1}$) is the thermal conductivity, ω (ml $\mathrm{mi}n^{-1}kg^{-1}$) is the volumetric blood perfusion, $Q_{m}$ (W kg^-1^) is the metabolic heat generation, *S* (W kg^-1^) is the SAR, which served as a source for the thermal simulations, and the subscript *b* denotes blood properties. Energy losses were modeled using a mix of convective and Neumann boundary conditions at the interfaces of tissue and water bolus (heat transfer coefficient: 40 $W{m^{-2}}^{\circ }C^{-1}$ ^48^). The initial temperature in tissues was set to 20°C (phantom) and 37°C (patient), while the temperature of the water bolus was set to 20°C. Note that the temperature elevation was defined relative to the initial temperature. For computational reasons, to solve Equation (2) forward in time, we spatially discretized the domain using a 3‐mm grid to obtain many coupled ordinary differential equations. These equations were solved with well‐known numerical integration techniques described in detail in our previous work.^14^ Additionally, the total computation time for thermal modeling was 4 min.

Table 1 lists the relative permittivity (ε_r_) and effective conductivity (σ) properties at 100 MHz,^49^, ^50^ and the thermal properties. The thermal properties of the phantom were assigned at baseline conditions,^51^ whereas the patient tissue properties were assigned at thermal stress conditions,^52^, ^53^ except for bone.^49^

**TABLE 1:** EM and thermal tissue properties of phantom and patient. The applicator‐shell, phantom‐shell, and phantom‐bone present the same properties as plastic

Material	$\varepsilon_{r}$(‐)	σ (S/m)	*c* (J/kg/°C)	*K* (W/m/°C)	*Q* (W/kg)	ρ (kg/m^3^)	*ω* (ml/min/kg)
Applicator‐shell	2.8	0.004	–	–	–	1180	–
Applicator‐water bolus	80.95	0.0026	–	–	–	1000	–
Phantom‐bone	2.8	0	1500	0.2	–	1600	–
Phantom‐inner	78.0	0.45	3630	0.64	–	1000	–
Phantom‐shell	2.8	0	1500	0.2	–	1600	–
Patient‐bone	15.3	0.0643	1313	0.32	0.15	1908	10
Patient‐muscle	66.0	0.708	3421	0.45	0.96	1090	300
Patient‐fat	12.7	0.0684	2348	0.21	0.51	911	200
Patient‐target	70.0	0.75	3950	0.51	–	1050	80

### 2.4 POD model reduction

The spatial discretization of the pelvic region's thermal model in the pelvic region results in an extensive state‐space system with approximately 10^5^ states^14^, ^54^ that is too complex to use in real‐time temperature predictions. The POD model reduction^36^ decreases the computational demand without compromising spatial resolution. This method reduces the model order by projecting the original model into a subspace generated by simulation snapshots.^54^ The snapshot matrix contains a set of observations obtained by precomputed thermal simulations. Equation (3) shows the formulation of the snapshot matrix (*Y*), where the columns define the snapshots and *k* denotes the number of snapshots.

(3) $$Y=\left[\begin{array}{cc} \xi \left(t_{1}\right) & \xi \left(t_{2}\right) \end{array}\;\begin{array}{cc} \text{…} & \xi \left(t_{k}\right) \end{array}\right]\in R^{n\times k}$$

Contrary to our previous work, the snapshot matrix was based on systematic impulse responses. This was based on the research conducted by Rowley et al. who showed that, because all reachable states can be written by a linear combination of impulse responses, the snapshot matrix created can describe any reachable state.^55^ Next, we used the singular value decomposition (SVD) to reduce the model order, which is defined as

(4) $$Y=U\Sigma V^{H},$$

where $U\in R^{n\times n}$,$\;\Sigma \in R^{n\times k}$ and $V\in R^{k\times k}$. Additionally, the columns of *U* represent orthogonal temperature modes from the snapshot matrix, whose importance is given by the corresponding single value in Σ. We truncated the SVD to obtain a low‐rank approximation of *Y* as given in Equation (4). To select the reduced model order without compromising the POD model accuracy, we truncated the singular values such that they included singular values that capture 95.5% of the energy in the snapshot matrix using the 2‐norm. For more details, see Eckart–Young–Mirsky theorem for the spectral norm.^56^ The first *m* columns of *U* are contained in $U_{m}$, that form the orthonormal basis for the approximation space of dimension *m*. Note that the orthonormal basis means that $U_{m}^{⊺}\;U_{m}=1$. Hence, the nodal state vector is now approximated by Equation (5).

(5) $$\xi \left(t\right)\approx U_{m}x\left(t\right),$$

where *x* is the reduced state.

Finally, the reduced system is obtained by applying Galerkin projection of the original model on the orthonormal basis $U_{m}$, and its formulation is given by

(6) $$\frac{dx\left(t\right)}{dt}=U_{m}^{T}\;AU_{m}x\left(t\right)+U_{m}^{T}Bq\left(t\right)+U_{m}h\left(t\right).$$

As a result, dominant effects will be extracted from this simulation‐based snapshot matrix to describe the 3D temperature distribution over time. The computation time to generate the POD‐reduced model was 10 min.

### 2.5 Kalman filter for recursive temperature estimation

The temperature estimation using the Kalman filter^24^ is summarized in two parts: (1) temperature prediction and (2) the combination of the obtained predicted temperature with the MR thermometry measurements. The first step predicts the temperature using the model based on the previous temperature estimate, and the settings of the RF‐applicator antennas. The predicted temperature at time *t* ($x_{t}^{p}$) and the prior estimate error covariance ($P_{t}^{p}$) are given by

(7) $$x_{t}^{p}=A_{t}\;x_{t-1\;}+\;B_{t}u_{t},$$

(8) $$P_{t}^{p}=A_{t}\;P_{t-1}A_{t}^{T}\;+\;Q,$$

where the superscript *p* indicates the model‐based prediction; $x_{t}^{p}$ denotes the predicted temperature at time *t*; $A_{t}$ and $B_{t}$ denote the reduced‐order discrete‐time system matrices obtained by projecting the time‐discretized dynamics on the precomputed POD subspace, which we obtained from multiple time simulations.^14^ Furthermore, $x_{t-1}$ denotes the estimated temperature at time *t* − 1; $u_{t}$ is the control input at time *t* using the settings of the RF applicator antennas, thus $B_{t}u_{t}$ is the heat load delivered by the RF applicator. Last,$\;P_{t}$ denotes the predicted state covariance, and Qis the process noise covariance, which is approximated by a diagonal covariance matrix and this defined as identity matrix.^14^

In the second step of the process, the Kalman filter combines the model‐based prediction with the filtered MR thermometry. To this end, at each time step, we compute the so‐called Kalman gain ($K_{t}$) that incorporates how much and where to trust the model‐based prediction and MR thermometry based on their respective covariance matrices. Equations (9), (10), and (11) summarize the measurement update equations.

(9) $$K_{t}=\left(P_{t\;}^{p}H^{T}\right)\;{\left(HP_{t}^{p\;}H^{T}+\;R\right)}^{-1},$$

(10) $$x_{t}=x_{t}^{p}\;+\;K_{t}\;\left(y_{t}\;-\;Hx_{t}^{p}\right),$$

(11) $$P_{t}=\;\left(I\;-\;K_{t}H\right)P_{t}^{p},$$

where *H* denotes the observation matrix that relates the model state to the measurement; $y_{t}$ denotes the filtered MR thermometry that represents MR thermometry after applying the filtering process and is described in the next paragraph; and $x_{t}\;$is the corrected state estimate that combines the model‐based estimate and the measurements. *R* denotes the measurement noise covariance, and it is approximated by a diagonal covariance matrix where each diagonal element represents the spatial variation around each MR thermometry voxel.^14^ Hence, this parameter is derived from the standard deviation of the MR thermometry. Note that *t* refers to all time points during the treatment or experiments, and only some of these time points refer to an MR thermometry update. The computation time for real‐time correction using temperature predictions should not exceed the interscan time (83 s). Furthermore, we chose a sampling time of 5 s; thus, every 5 s temperature estimate was available. More details on implementing the POD–Kalman filter can be found in our previous work.^14^

**FIGURE 2:**
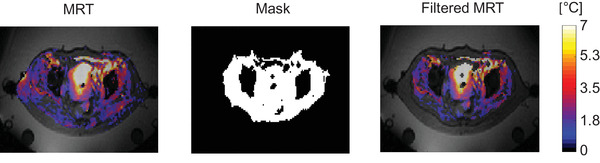
Temperature distributions of a representative patient after and before applying the mask created during the filtering process. The subcutaneous fat is not included since this region was used for B0 field drift correction

Furthermore, MR thermometry was filtered such that corrupted data were excluded to avoid incorporating these corrupt measurements into the Kalman filtering algorithm. As presented in Figure 2, the MR thermometry after the data exclusion is defined as filtered MR thermometry, and as mentioned before, the filtered MR thermometry was used for recursive temperature estimation. We assumed data were unreliable when the difference between two subsequent MR thermometry acquisitions (ΔMRT) and subsequent predictions (ΔState) showed a significant discrepancy. We defined the prediction error as Equation (14).

(12) $$\Delta MRT\;=\;\Delta T\left(t\right)-\Delta T\left(t-1\right),$$

(13) $$\Delta State\;=x_{t}^{p}\;-x_{t-1}^{p},$$

(14) Predictionerror=ΔMRT−ΔState.

We excluded data points with a prediction error higher than 7°C and lower than −4°C. Additionally, we considered that between two subsequent MR thermometry acquisitions, there should not be a decrease higher than 3°C; thus, any ∆MRT lower than −3°C was filtered out of the final filtered MR thermometry. Figure 2 presents the mask created from the above thresholds. Moreover, regions that exceeded the above thresholds were masked out from the MR thermometry (black regions). Consequently, these masked regions were not used in the Kalman filter algorithm.

### 2.6 Experiment and treatment setup

Intraluminal thermometry and two different MR scans were taken during experiments and treatments: a high‐resolution scan and MR thermometry. The high‐resolution scan was a T1‐weighted MR image used to generate the patient model, position verification, and identify the catheters containing Bowman probes. The sequence used for the high‐resolution scan was a DEGRE sequence with the following parameters: echo times, TE = 4.8 and 9.6 ms; repetition time, TR = 120 ms; 25 axial slices; slice thickness = 1 cm; FOV = 50 cm × 50 cm; acquisition matrix = 128 × 128; reconstruction matrix = 256 × 256; flip angle = 70°; scan time = 136 s. MR thermometry scans were taken to measure the 3D temperature elevation. All heating experiments and hyperthermia treatments were conducted between 2017 and 2019.

#### 2.6.1 Heating phantom experiments

For phantom 1, we used two different antenna settings focusing at (*x*,*y*,*z*) = (3,0,0) cm and (*x*,*y*,*z*) = (−3,0,0) cm. The total power applied in all experiments was 600 W. In the experiments using phantom 1, one scan was taken before heating, and two sets of six automated MR thermometry scans were taken during heating. For each set of MR thermometry scans, one of the two foci was used. Regarding the two experiments using phantom 2, we applied only one set of antenna settings, with the energy deposition focus at the center of the phantom at (*x*,*y*,*z*) = (0,0,0) cm. In all experiments, the water in the water bolus was not circulated during heating to prevent artifacts. After the power was turned off, extra scans were taken to incorporate disturbances. In one experiment, three MR thermometry scans were taken while the door of the MR room was open, and sequentially, at the end of the experiment, an extra MR thermometry scan was taken with water circulation on. In all other heating experiments, water circulation from the water bolus was the only disturbance included. Hereto, water circulation was turned on in the last MR thermometry scans: One extra scan was taken in four experiments; two extra scans were taken in two experiments, and three extra scans were taken in the one experiment that also included opening the door.

**FIGURE 3:**
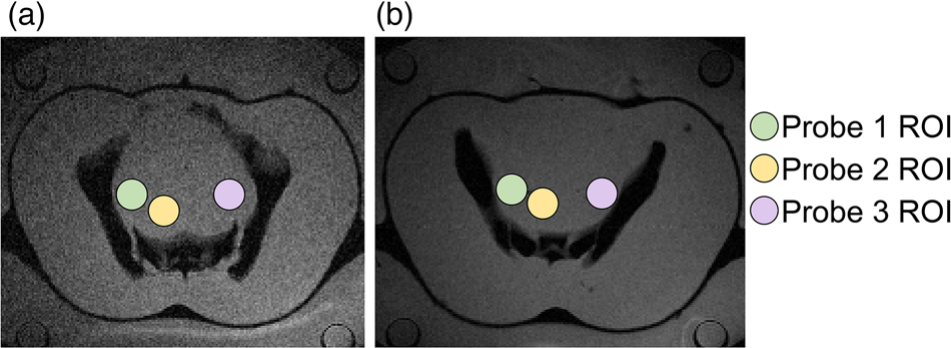
Axial T2‐weighted MR images of the two phantoms are shown, along with the location of the Bowman probes and the corresponding ROIs. Phantom 1 is presented in (a) and phantom 2 is presented in (b)

Figure 3 presents the location of the Bowman probes and the corresponding ROI, with an area of 1.5 cm^2^. The intraluminal measurements acquired continuously correspond to the middle slice of the simulation and MR thermometry FOV.

#### 2.6.2 Hyperthermia treatments

**FIGURE 4:**
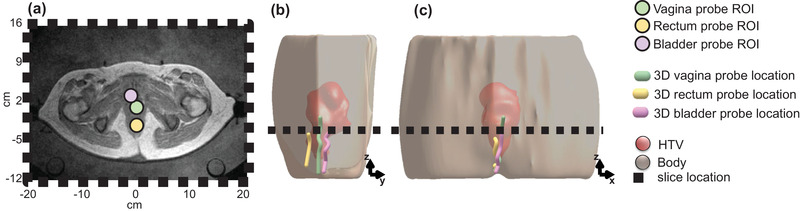
Axial T2‐weighted MR image (a) of the pelvic region is shown along with the Bowman probes ROIs: vagina, rectum, and bladder. The location and path of the probes in the 3D model is shown in the sagittal (b) and coronal (c) view. The dotted line represents the axial location of the T2‐weighted MR image

MR thermometry scans were taken approximately every 10 to 20 min following the clinical protocol. A specific treatment plan was made for each patient to define the settings that should be applied to the antennas.^44^, ^47^, ^57^ Treatment settings for power and phase were not modified during treatment since there were no patient complaints in the included treatments, and the temperature registered by the probes did not exceed 43°C. The average heating power applied during treatment was between 800 W and 1000 W. As shown in Figure 4, Bowman probes were inserted into closed tip catheters placed in the bladder, vagina, and rectum. The intraluminal measurements were acquired along with the catheters. This thermal mapping was performed every 5 min with a step size of 1 cm (Figure 4). Considering all intraluminal locations, the thermal mapping range was 8.5 cm ± 2.8 cm.

**FIGURE 5:**
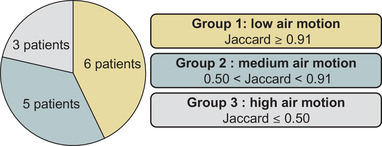
Schematic representation of the three groups of patients according to the amount of intestinal gas motion. Each group included the patients with a Jaccard coefficient (Jaccard) between the corresponding interval

Our previous study^22^ showed that the Jaccard coefficient (Jaccard ≥ 0.91) between two baseline scans is predictive of MR thermometry accuracy. The Jaccard coefficient was used to quantify the level of intestinal gas motion and, consequently, the resulting susceptibilities artifacts. To characterize the improvement in MR thermometry accuracy, we organized the 14 patients (14 hyperthermia treatment sessions) into three groups according to the Jaccard coefficient measured at the beginning of the treatment (Figure 5). Hence, the patient group with a lower Jaccard coefficient represents the treatments where the MR thermometry measurements presented strong susceptibility artifacts due to the high level of intestinal gas motion.

### 2.7 Statistical analysis

For each treatment and experiment, accuracy is expressed by the mean absolute error (MAE). This was calculated to quantify the degree of closeness of the filtered and measured temperature change to the true temperature change formulated in Equation (15).^11^, ^58^, ^59^ In addition, we calculated the standard deviation of error (SDE) to evaluate MR thermometry and POD–Kalman filter consistency. The formulation of SDE is presented in Equation (18). We considered an MAE ≤1°C^11^, ^58^, ^59^ and an SDE ≤0.5°C^11^ as acceptable.

(15) $$MAE\;=\frac{1}{n}\;\sum_{j\;=\;1}^{n}\;\left|{\bar{T}}_{X_{ROI},\;j}\;-\;{\bar{T}}_{probe_{ROI},\;j}\right|,$$

(16) $$\varepsilon_{j}=\;{\bar{T}}_{X_{ROI},\;j}\;-\;{\bar{T}}_{probe_{ROI},\;j},$$

(17) $$\bar{\varepsilon }=\frac{1}{n}\;\sum_{j\;=\;1}^{n}\varepsilon_{j},$$

(18) $$SDE^{2}=\frac{1}{n-1}\;\sum_{j\;=\;1}^{n}{\left(\varepsilon_{j}\;-\;\bar{\varepsilon }\right)}^{2},$$

where subscript *X* is the POD–Kalman filtered temperature or filtered MR thermometry, ${\bar{T}}_{X_{ROI},\;j}$ is the average temperature in the ROI; ${\bar{T}}_{probe_{ROI},\;j}$ is the average temperature measured by the Bowman probes, and *n* are measured time points; $\varepsilon_{j}$ is the error between the method and the average temperature measured by the Bowman probes; and $\bar{\varepsilon }$ is the mean error value over the measured time points. Note that the voxels included in each ROI were weighted uniformly; thus, all voxels presented the same weight.

The MAE and SDE were described as mean ± standard deviation. We compared the MAE and SDE using Mann–Whitney U‐test followed by a post hoc Tukey's test to analyze if a statistically significant difference could be shown between the POD–Kalman filter and MR thermometry. A *p*‐value of 0.05 was considered to be statistically significant. First, we considered the average MAE and SDE, including all probes, and compared the two methods. Second, we looked into the differences between the two methods for each patient group and probe location. This evaluation was conducted for all patients (14 patients) and heating experiments (eight experiments).

## 3 RESULTS

### 3.1 Phantom heating experiments

**FIGURE 6:**
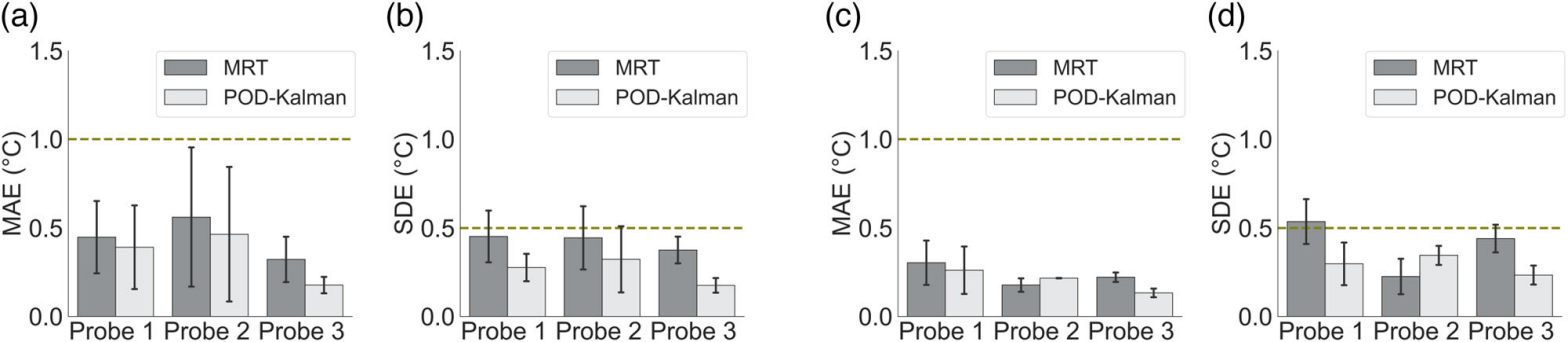
MAE and SDE of filtered MR thermometry and Kalman filtered temperature prediction in each probe location and phantom. (a) and (b) present the MAE and SDE from phantom 1, and (c) and (b) show the MAE and SDE from phantom 2. The error lines show the standard deviation from all experiments, and the height of the bars represents the average within the experiments. The dashed green line represents the acceptable MAE and SDE. The bar graphs in (a) and (b) are concerning to six experiments, while the bar graphs in (c) and (d) are regarding to two experiments

The total average ± standard deviation of the measurement noise covariance (*R* from Equation 9) was 0.21°C ± 0.05°C for all experiments. Note that the noise covariance reported does not include the regions of bone. Figure 6 shows MAE and SDE for probe locations and phantoms within the ROIs. The SDE results showed that the POD–Kalman filtered temperatures were consistently closer to the probe measurements. Even though the SDE after the POD–Kalman filter was significantly better than the filtered MR thermometry (*p*‐value < 0.01), there was no significant improvement in MAE (*p*‐value = 0.09).

Overall, the MAE values acquired for both methods met the threshold for acceptance. The average MAE in all phantoms and all locations were 0.39°C ± 0.27°C (filtered MR thermometry) and 0.31°C ± 0.27°C (POD–Kalman filtered temperature). The average SDE was 0.42°C ± 0.15°C (filtered MR thermometry) and 0.27°C ± 0.13°C (POD–Kalman filtered temperature). POD–Kalman filtering also effectively reduced the SDE to the acceptable limits for the three probe locations in both phantoms.

Table 2 shows the MAE and SDE between the probe reading and each method in the scanning times that included disturbances to evaluate the impact of the applied disturbances. We observed that the disturbances did not affect the performance of the POD–Kalman filter since it was consistently better than MR thermometry.

**TABLE 2:** MAE and SDE of all scanning times where disturbances were applied (water circulation and the MR room open door). Note that these values are expressed by the mean ± standard deviation, and for each parameter, we indicate the statistical significance

	MRT	POD—Kalman	*p*‐value
MAE	1.23 ± 0.53°C	0.51 ± 0.20°C	0.04
SDE	1.01 ± 0.45°C	0.53 ± 0.31°C	<0.01

### 3.2 Hyperthermia treatments

#### 3.2.1 Evaluation between probe locations

The percentage of temperature information available in the filtered MR thermometry was approximately 50% for all probe locations, while for the POD–Kalman filtered temperature, all temperature information was used (100%). The amount of information that remained from the filtering process was quantified using the resulted Mask from Figure 2.

**FIGURE 7:**
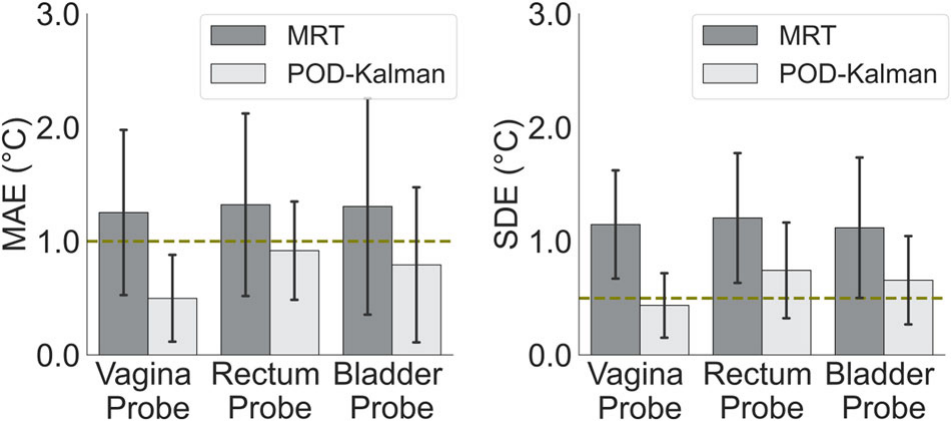
MAE and SDE of filtered MR thermometry and POD–Kalman filtered temperature in each anatomical probe location (vagina, rectum, and bladder). The error lines show the standard deviation from all experiments, and the height of the bars represents the average. The dashed green line represents the acceptable MAE and SDE. The MAE and SDE are calculated for the total patients included in this study (*n* = 14)

Figure 7 and Table 3 present the evaluation regarding MAE and SDE for each anatomical probe location. As observed in Figure 7, the POD–Kalman filtered temperature presented an MAE under the acceptable threshold for all probe locations, while MAE acquired from filtered MR thermometry was always higher than 1°C. The number of patients whose POD–Kalman filtered temperatures presented an MAE lower than 1°C was 12 patients in the vagina probe, 10 patients in the bladder probe, and 8 patients in the rectum probe. For all probe locations, only half of the patients (seven patients) had an MAE lower than 1°C for the filtered MR thermometry. Even though the MAE from POD–Kalman filtered temperature was better in all anatomical probe locations, the improvement was statistically significant only for measurements in the vagina. For all anatomical probe locations (Figure 7), SDE from POD–Kalman filtered temperatures was significantly better than from filtered MR thermometry (*p*‐value < 0.05).

**TABLE 3:** MAE and SDE are expressed by the mean ± standard deviation. These were calculated for three different anatomical locations: V‐vagina, R‐Rectum, and B‐Bladder. The evaluation parameters concern all patients included in this study (14 patients)

	MAE (°C)			SDE (°C)		
	MRT	POD–Kalman	*p*‐value	MRT	POD–Kalman	*p*‐value
V	1.25 ± 0.75	0.50 ± 0.40	<0.01	1.15 ± 0.49	0.44 ± 0.29	<0.01
R	1.32 ± 0.83	0.92 ± 0.45	0.28	1.20 ± 0.59	0.74 ± 0.44	0.03
B	1.31 ± 0.99	0.79 ± 0.71	0.06	1.12 ± 0.64	0.66 ± 0.40	0.04

POD–Kalman filter reduced MAE by 43% for all patients since we found 0.74°C (POD–Kalman filtered temperatures) and 1.29°C (filtered MR thermometry). Even though POD–Kalman filtering strongly reduced SDE and MAE, the requirements were not met in all patients (Figure 7). The average SDE was 1.16°C ± 0.56°C (filtered MR thermometry) and 0.61°C ± 0.40°C (POD–Kalman filtered temperature). Even though the POD–Kalman filter does not meet the requirements in all patients and locations, we believe that this method outperformed the efficacy of MR thermometry. Overall, the POD–Kalman filtered temperatures were more consistent and closer to the probe readings than MR thermometry, with a *p*‐value < 0.001 for MAE and SDE. Though, we observed that the performance of the POD–Kalman filter was ambiguous in the rectum location since the requirements for SDE and MAE were only met in six and eight patients, respectively.

#### 3.2.2 Evaluation between patient groups

The percentage of temperature information available in the filtered MR thermometry near the probe was 63% (group 1: little intestinal gas motion), 44% (group 2: medium intestinal gas motion), and 36% (group 3: high intestinal gas motion). The number of patients whose filtered MR thermometry showed more than 50% of temperature information near the probe was five patients, two patients, and zero patients for group 1, group 2, and group 3, respectively. Note that the amount of information not filtered out from the MR thermometry was quantified using the mask acquired from the filtering process (Figure 2). Since no filtering was applied in the estimated temperatures, the percentage of information in POD–Kalman filtered temperature was 100% in probe regions.

**FIGURE 8:**
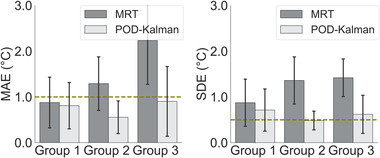
MAE and SDE of filtered MR thermometry and POD–Kalman filtered temperature in each patient group: 1—group 1 (low intestinal gas motion), 2—group 2 (medium intestinal gas motion), and 3—group 3 (high intestinal gas motion). The error lines show the standard deviation from all experiments, and the height of the bars represents the average. The dashed green line represents the acceptable MAE and SDE. The MAE and SDE are calculated for the total patients included in this study (*n* = 14)

Figure 8 and Table 4 confirm the relation between MAE from filtered MR thermometry and the Jaccard coefficient since patients with higher Jaccard coefficients have a lower MAE.^22^ Even though more motion was measured in patients from group 2 and group 3, the POD–Kalman filter reduced MAE to below the acceptable threshold for most patients in all groups.

**TABLE 4:** MAE and SDE are expressed by the mean ± standard deviation. These were calculated for the three different groups that were organized according to the intestinal gas motion: 1—group 1 (low intestinal gas motion), 2—group 2 (medium intestinal gas motion), and 3—group 3 (high intestinal gas motion). The evaluation parameters aconcern all patients included in this study (14 patients)

	MAE (°C)			SDE (°C)		
	MRT	POD–Kalman	*p*‐value	MRT	POD–Kalman	*p*‐value
1	0.88 ± 0.57	0.81 ± 0.52	0.81	0.87 ± 0.53	0.71 ± 0.47	0.17
2	1.29 ± 0.61	0.55 ± 0.37	<0.01	1.36 ± 0.53	0.49 ± 0.21	<0.01
3	2.23 ± 1.02	0.90 ± 0.81	<0.01	1.41 ± 0.44	0.61 ± 0.44	<0.01

The SDE acquired from filtered MR thermometry showed that these measurements were not consistently accurate in any group. Even though the SDE for group 1 was lower than in other groups, this value was still 0.37°C higher than the acceptable threshold. For the POD–Kalman temperatures, the SDE was below the acceptable threshold only for group 2. Although the results from group 1 showed improvement, this was not significant when using the POD–Kalman filter in both MAE (*p*‐value = 0.81) and SDE (*p*‐value = 0.17). Additionally, for group 2 and group 3, we observed that the POD–Kalman filter performed significantly better in both evaluation parameters. As observed in Table 4, in both parameters, the resulting *p*‐value was below 0.01 for group 2 and group 3.

We quantified the percentage of patients whose MAE was below the acceptable thresholds for each group and method in at least two probe locations. As presented in Table 5, in group 2 and group 3, we observed an improvement when using the POD–Kalman filter as more patients presented an MAE according to the acceptable limits.^11^ Overall, the POD–Kalman filter allowed thermal dosimetry during treatment in most patients (12 out of 14 patients). In contrast, MR thermometry would enable thermal dosimetry in only half of the patients presented (7 out of 14 patients).

**TABLE 5:** Number of patients from each group where the temperatures acquired in at least two probe locations presented an MAE ≤ 1°C

Group	MRT	POD–Kalman	Improvement
1 (*n* = 6)	5	5	+0 patients (0%)
2 (*n* = 5)	2	4	+2 patients (43%)
3 (*n* = 3)	0	3	+3 patients (100%)

#### 3.2.3 Temperature evolution during treatment

For calculating POD–Kalman filtered temperatures, the measurement noise covariance (*R* from Equation 9) was calculated for each patient. The same procedure was done for patients where the bone regions were not included in calculating the noise covariance. Hence, we calculated the mean covariance, and we observed that for all patients, the total average ± standard deviation was 3.11°C ± 1.08°C. Furthermore, the amount of available information in the filtered MR thermometry was limited due to artifacts. The mask (Figure 2) created in the filtering process was used to define the filtered MR thermometry and, consequently, the amount of information used in the POD–Kalman filter. Overall, over the entire filtered MR thermometry FOV, only 41% of this volume was used in the POD–Kalman filter.

**FIGURE 9:**
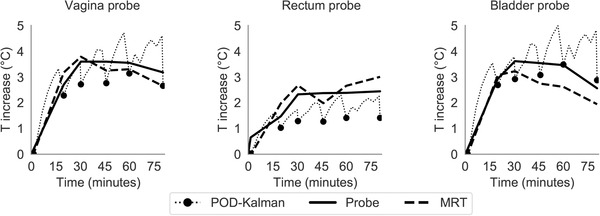
Temperature profile in the three probe locations during treatment. The temperature profiles correspond to a patient from group 1. The dotted line represents the Kalman prediction between MR thermometry updates

Figure 9 displays the temperature evolution at each anatomical probe location for a patient in group 1. The results show that the temperature from filtered MR thermometry measurements did not vary substantially. Although the POD–Kalman filtered temperature presented a closer agreement in the bladder, the predicted temperature in the rectum and vagina was lower than the registered intraluminal temperature. During treatment, the POD–Kalman filtered temperature underestimated the temperature by an average of 0.46°C, while the filtered MR thermometry underestimated the temperature by 0.13°C (Figure 9). In addition, the POD–Kalman filtered temperature tends to have a lower temperature even when the filtered MR thermometry measurements gave higher temperatures (dotted line in Figure 9). This contradiction is because the Kalman filter estimates a temperature distribution consistent with the filtered MR thermometry over the total patient volume (MRT FOV). Hence, a local comparison at the probe locations might yield counterintuitive results. Due to the global nature of the Kalman filter, removing corrupt data is crucial as the effects of these can propagate to distant regions.

**FIGURE 10:**
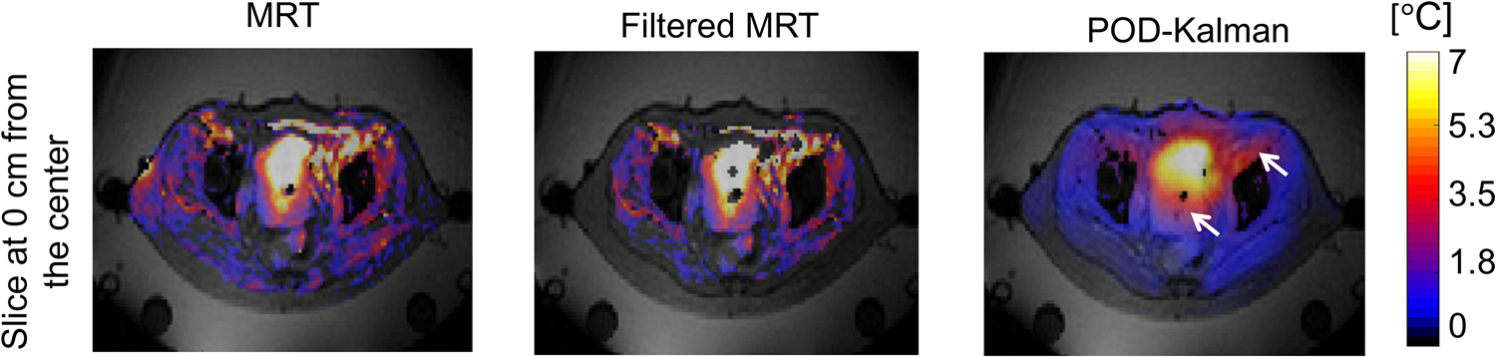
Temperature distributions of a patient from group 1 after 1 h of treatment: MR thermometry (MRT), filtered MR thermometry (filtered MRT), and POD–Kalman filtered temperature (POD–Kalman). The white arrow indicates the regions of the POD–Kalman filtered temperature where the temperature is underestimated compared to the filtered MR thermometry. Note that MRT is the MR thermometry measurements without any filtering

Figure 10 shows the temperature distribution acquired by MR thermometry, filtered MR thermometry, and POD–Kalman filtering after 60 min of treatment. The white arrows point at the regions where POD–Kalman filtered temperature is lower despite the temperature increase in the model and filtered MR thermometry. In addition, Figure 10 shows that the POD–Kalman filtered temperature is smoother than the filtered MR thermometry measurements. Regions of subcutaneous fat were filtered out since they were used for drift correction. In the MR thermometry in Figure 10, we observed an increase in temperature in the subcutaneous fat that was filtered out. This increase was likely due to an artifact since no complaint by the patient was reported during this hyperthermia treatment.

**FIGURE 11:**
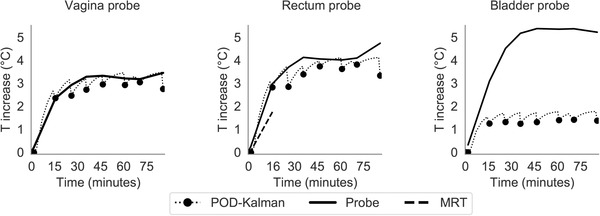
Temperature profile during treatment in the three probe locations. The temperature profiles correspond to a patient from group 3. The dotted line represents the Kalman prediction between MR thermometry updates. The filtered MR thermometry (MRT) curve is not continuous over time due to the absence of reliable measurements in the region around the probe

Contrary to the patient from group 1 (low intestinal gas motion), the filtered MR thermometry from a patient of group 3 (high intestinal gas motion) was substantially corrupted by noise and motion. Figure 11 shows a close agreement between POD–Kalman filtered temperature and probe measurements in the rectum and vagina. However, we observed that the POD–Kalman filter underestimates the temperatures obtained during the treatment in the bladder location by 2.8°C. This difference is possibly due to a bladder modeling mismatch. During treatment, there were no reliable MR thermometry measurements, as observed by the discontinuity of the MR thermometry curve in all probe locations. Only in the first scanning point reliable MR thermometry measurements were available.

**FIGURE 12:**
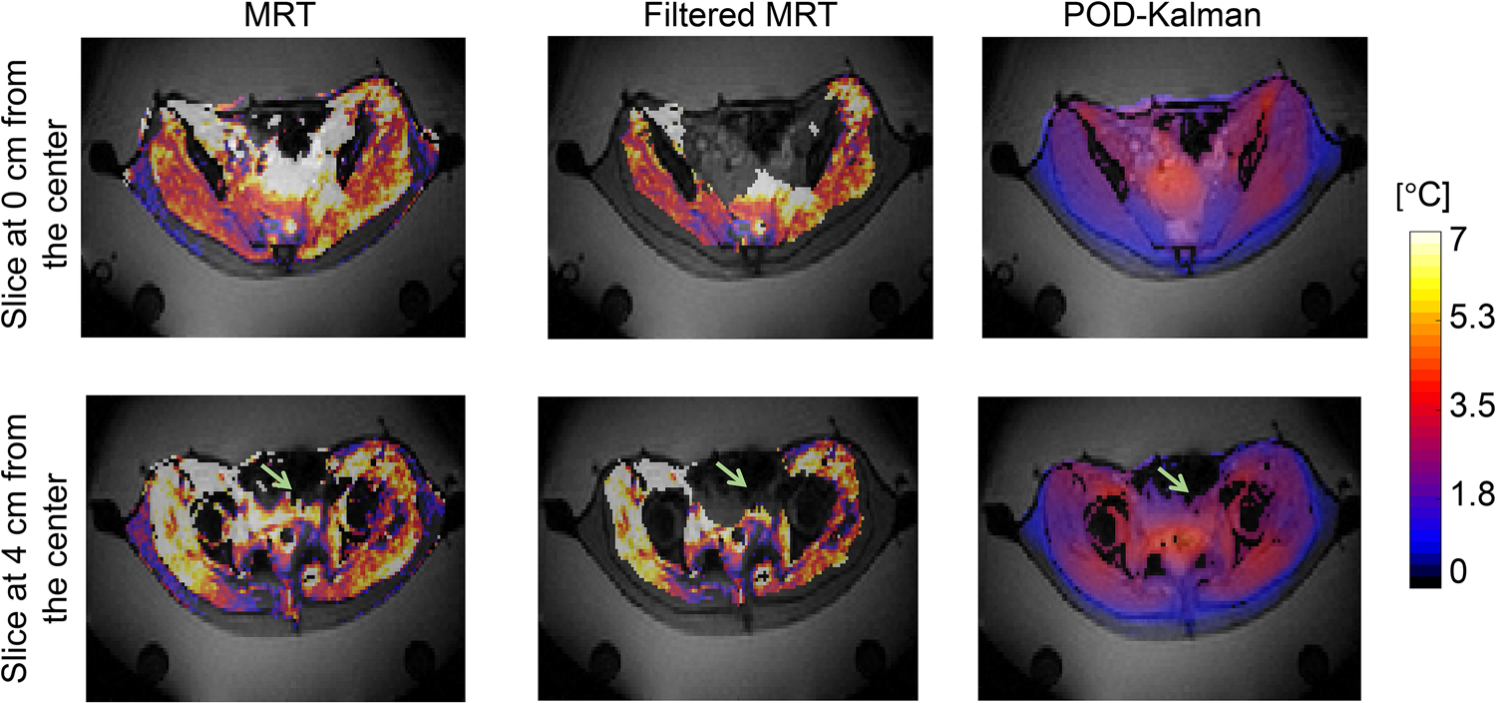
Temperature distributions of a patient from group 3 after 1 h of treatment: MR thermometry (MRT), filtered MR thermometry (filtered MRT), and POD–Kalman filtered temperature (POD–Kalman). The light green arrow points to where the bladder probe is located. Note that the MRT is MR thermometry measurements without any filtering

Figure 12 illustrates the temperature distribution in two different locations in the coronal plane. The underestimation of the POD–Kalman filtered temperature observed in the bladder is due to urine that was not modeled or identified but assigned as muscle (Figure 12). In addition, this area presented a high intestinal gas motion, which made the information from filtered MR thermometry in that region inexistent.

## 4 DISCUSSION

In this study, we evaluated the performance of POD‐based reduced models for recursive temperature estimation using a Kalman filter in several phantom experiments and patient data. Even though there was no significant improvement in MAE for phantoms, SDE was significantly lower in the POD–Kalman filtered temperature (Figure 6). The high MAE and SDE values in probe 2 (phantom 1) are probably related to the heat diffusion from both foci to the center region and the inhomogeneity of the material inside phantom 1. Furthermore, we verified that the POD–Kalman filter was insensitive to the disturbances during the experiment (Table 2). Since near disturbances, MR thermometry measurements were unreliable, POD–Kalman filter relied only on the POD model. Overall, we observed that the MAE in both methods was satisfactory and agreed with previous studies.^9^, ^14^ However, in all phantom experiments, the complexity of the thermal model was low, and the noise covariance of the MR thermometry was very low.

We observed that the POD–Kalman filtering improved MAE and SDE compared to MR thermometry regarding the patient data and considering all probe locations. SDE was statistically improved at each anatomical probe location when using the POD–Kalman filter. In the rectum and bladder, the improvement by the POD–Kalman filter was not statistically significant in MAE (Figure 7). The model predictions for these locations are prone to uncertainties in the modeling, and the measurements are affected by anatomy changes (Figure 12). First, organ movement leads to incorrect spatial temperature reference, which will cause inaccuracies in the MR thermometry measurements.^12^, ^22^, ^60^, ^61^ Second, the POD model is based on the patient's position at the start of treatment; thus, the changes in patient anatomy during the treatment are not considered. This was observed in the patient from group 3, whose filtered MR thermometry and POD–Kalman filtered temperature were not physically accurate in the bladder region. The difference between the temperatures registered in the probe and POD–Kalman filtered temperatures suggests a model mismatch (Figure 11), which was not corrected by the filtered MR thermometry due to the significant inaccuracies in that region (Figure 12). Henceforth, the POD–Kalman filter will not yield satisfactory results when these phenomena are not physically captured by either the modeling or MR thermometry.

Several studies have shown that MR thermometry assessment in the pelvic region is challenging.^19^, ^22^, ^62^ Our previous study showed that the amount of intestinal gas motion at the start of the treatment was an MR thermometry accuracy predictor and that a higher level of motion caused stronger susceptibility artifacts. Hence, patients with a high intestinal gas motion would not be amendable for reliable MR thermometry acquisition.^22^ In the current study, three patient groups were defined according to the level of intestinal gas motion (Jaccard coefficient). The aim was to assess if this method could compensate for the missing or unreliable MR thermometry measurements and, at the same time, still estimate temperature accurately based on the POD model.

Based on the defined requirements for MR thermometry during hyperthermia,^11^ we demonstrated that the average MAE from POD–Kalman filtered temperature was under the acceptable thresholds in all patient groups (MAE ≤ 1°C). The results proved that the POD–Kalman filter could estimate reliable temperature for patients with a low Jaccard coefficient. Furthermore, our findings showed that the MAE and SDE are not significantly improved when intestinal gas motion is low (group 1) since the MR thermometry measurements are already satisfactory. On the other hand, despite the unreliable filtered MR thermometry measurements (Figure 12), the POD–Kalman filter significantly improved the MAE in the presence of medium (group 2) and high (group 3) intestinal gas motion by 57% and 60%, respectively. In group 2 and group 3, the percentage of patients with reliable temperature distributions was also higher than if only the filtered MR thermometry were used. In these groups, the number of filtered MR thermometry data points remained less than 50%. These results indicate that the POD–Kalman filter would allow thermal dosimetry for patients who are not amendable for accurate MR thermometry acquisition (Table 5).

POD–Kalman filter performance was generally satisfactory; however, we also observed that this method could not accurately estimate the temperature in some cases. One of the reasons is that its effectiveness relies on the accurate representation of the heating process in the modeling. The patient model used was based on the anatomy and position at the start of the treatment. Hence, any change during the treatment was not considered during the estimation process. Also, there are significant uncertainties in the thermal parameters of each tissue,^45^ reducing the accuracy of thermal modeling used to generate the POD library. Therefore, the model's inaccuracy and the possibility of nonmodeled disturbances influence the performance of the POD–Kalman filter and MR thermometry filtering process. Hence, the improvement of the thermal model is subject to further research. Also, we observed in the results from the patient from group 1 (Figure 9 and Figure 10) that the POD–Kalman filtered temperatures were lower, even though the temperature in the POD model and MR thermometry were high. This contradiction is probably due to the imperfect filtering of corrupt data. In order to solve the latter problem, we recommend further research in improving the MR thermometry filtering by selecting only reliable regions. Hence, as a first step, we propose to track the anatomical changes over the treatment using the anatomic MR images and select the regions that showed more stability. Also, we propose the use of variational data assimilation,^63^ which optimizes an initial state of a dynamical system using a predefined objective. This function considers the amount of noise introduced into the system and the mismatch between simulated and observed variables. In addition to the improvements referred to before, we believe that further research in new MR sequences and approaches is needed to improve MR thermometry acquisition and performance. In the meantime, until the filtering, thermal modeling, and MR thermometry performance are improved, we advise that the estimated temperatures in clinical practice are supported by data from intraluminal probes.

Other studies have shown the benefit of combining Kalman filtering with MR thermometry using patient data,^29^, ^30^ and found that temperature monitoring could be improved by this approach. However, these studies were based on data from HIFU treatments, in which the heating volumes are considerably smaller and modeling complexity is therefore lower. Our study is the first in showing the feasibility of POD–Kalman filtering for patient data of RF hyperthermia treatments. Our results also indicate that the combined method provides a systematic improvement compared to MR thermometry alone. We established that POD–Kalman filtering could be used in the clinic as complementary information since it enables real‐time performance. Furthermore, the proposed POD–Kalman method presents high temporal resolution, enabling the development of MR sequences, real‐time filtering, and corrections.

## 5 CONCLUSION

For MR‐guided RF hyperthermia, the POD–Kalman filter approach showed significant improvements in the accuracy of MR thermometry in heating experiments and for data from patient treatments. For phantoms, relevant improvements were observed, but the significance could not be demonstrated with the number of experiments performed since MR thermometry is already highly accurate in phantoms.

In patients, we also observed relevant improvements by using the POD–Kalman filter. Especially in patients with medium and high intestinal gas motion, the POD–Kalman filter reduced the MAE: 57% (Jaccard coefficient = 0.5–0.91) and 60% (Jaccard coefficient < 0.5). The POD–Kalman filter was especially beneficial in patients whose MR thermometry is unreliable. In addition, POD–Kalman has the advantage that it also provides estimates between MR thermometry scans. The proposed POD–Kalman method is a promising approach that can already be used to provide additional information during MR‐guided RF hyperthermia treatment since this allows real‐time temperature estimation. Further advances in modeling and MR thermometry filtering and performance are necessary to meet the requirements for all patients. Hence, we believe that our results are a great motivation for improving thermal modeling and the MR thermometry acquisition.

## CONFLICT OF INTEREST

The authors declare no conflict of interest. The funders had no role in the study's design, in the collection, analyses, or interpretation of data; in the writing of the article; or in the decision to publish the results.

## Data Availability

The data that support the findings of this study are available from the corresponding author upon reasonable request.
